# Scoring the capillary leak syndrome: towards an individualized gradation of the vascular barrier injury

**DOI:** 10.1186/s13613-022-01000-0

**Published:** 2022-03-21

**Authors:** Thibaut Belveyre, Can Ince, Philippe Guerci

**Affiliations:** 1 Department of Anesthesiology and Critical Care Medicine, Institut Lorrain du Coeur Et Des Vaisseaux, University Hospital of Nancy, Rue du Morvan, 54511 Vandoeuvre-les-Nancy, France; 2 INSERM U1116, University of Lorraine, Nancy, France; 3 Department of Intensive Care Medicine, Laboratory of Translational Intensive Care, Erasmus MC University Hospital Rotterdam, Rotterdam, The Netherlands

## Comment

Wollborn et al*.* nicely described a new scoring system for non-invasive detection of capillary leakage in critically ill patients [1]. The authors are commended for their efforts in elucidating and refining the definition of the so-called Capillary Leak Syndrome (CLS). To date, there is no consensus definition of this phenomenon related to the tendency of fluid escaping quickly from the vascular space. CLS is thought to be a complex mix of endotheliopathy, glycocalyx shedding, vascular hyporesponsivness, disruption of cell-to-cell junctions, and microcirculatory flow and oxygenation alterations consequently leading to fluid accumulation within the interstitium.

Some important points remain of concern in their study. First, the investigated population mostly included postoperative patients, where post-operative events are largely related to events occurring during surgery [2]. The authors did not report enough detail regarding the duration of surgery, the type of fluids administered (including transfusion), whether fluid administration was goal-directed (hemodynamic monitoring), and the intraoperative requirement of vasopressors or inotropes [2]. These parameters may have had a direct consequence on the subsequent development of CLS. Indeed, patients in shock in their study are more likely to develop CLS.

Second, without challenging the expertise of the physicians assessing CLS, we wonder whether the criteria used for the diagnosis of CLS are specific to increased vascular permeability or may simply refer to osmotic pressure decrease, fluid accumulation or venous congestion occurring invariably in the postoperative course of these patients. Indeed, even in healthy subjects, fluid loading with 1.5L of crystalloids will inevitably accumulate and expand the interstitium [3]. If the objective was to characterize an increased vascular permeability, other techniques such as the albumin transudation rate or the radiolabeled albumin plasma volume determination should have been used.

Third, the authors identified several biomarkers of endothelial injury: glycocalyx degradation (Syndecan-1) and endothelial cell instability (Angiopoietine-2, ICAM-1). While glycocalyx shedding can occur independently of increased vascular permeability [4], other biomarkers such as VE-cadherin, which is considered an essential molecule of intercellular junction, was not integrated in the multivariate analysis and elaboration of the score. Because it may be important to graduate the injury to the vascular barrier, it is essential to integrate all the components forming the vascular barrier at different levels, and the hydrostatic and oncotic forces that control transendothelial fluid sieving (Starling’s equation). Besides, in their study, no direct (intravital microscopy) or indirect measurements of the microcirculation (apart from lactate) and microvascular reactivity were performed. The interpretation of those surrogate markers of CLS/vascular permeability is central to understand the mechanism underlying this phenomenon. Thus, evaluation of the respective contributions to barrier function of the key players according to severity score could have been interesting. This approach could likely (1) allow a gradation of vascular injury according to the clinical severity and (2) detect thresholds which are clinically relevant to prevent CLS or to initiate a personalized treatment.

Wollborn et al. paved the way towards a more comprehensive multifaceted evaluation of endothelial barrier and vascular permeability and injury, encompassing biomarkers and clinical signs at the bedside. Whether CLS is an independent entity and not just a consequence of the severity of a disease/inflammation and intensity of the resuscitation remains to be elucidated [5].

## Data Availability

Not applicable.

## Abbreviations

CLS: Capillary leak syndrome
BIA: Bioelectrical impedance
